# Digestibility and metabolism of copper in diets for pigs and influence of dietary copper on growth performance, intestinal health, and overall immune status: a review

**DOI:** 10.1186/s40104-020-00533-3

**Published:** 2021-01-11

**Authors:** Charmaine D. Espinosa, Hans H. Stein

**Affiliations:** 1 Department of Animal Sciences, University of Illinois, Urbana, 61801 USA; 2 Division of Nutritional Sciences, University of Illinois, Urbana, 61801 USA

**Keywords:** Copper, Copper nutrition, Intestinal health, Metabolism, Pharmacological concentrations, Pigs

## Abstract

The current contribution reviews absorption and metabolism of copper (Cu), Cu deficiency, Cu toxicity, Cu bioavailability, and effects of pharmacological levels of Cu on growth performance and intestinal health of pigs. Copper is a micro mineral involved in metabolic reactions including cellular respiration, tissue pigmentation, hemoglobin formation, and connective tissue development. Copper is mostly absorbed in the upper gastrointestinal tract, particularly in the duodenum, but some Cu is absorbed in the stomach. One way to evaluate the efficacy of sources of Cu is to measure relative bioavailability where responses include tissue concentrations of Cu, concentrations of metalloproteins, and enzymatic activity of animals fed test diets containing graded levels of Cu. The requirement for Cu by pigs is 5 to 10 mg/kg diet, however, Cu can be included at growth-promoting levels (i.e., 75 to 250 mg/kg diet) in diets for weanling and growing pigs to reduce post-weaning diarrhea and improve growth performance. The consistently observed improvement in growth performance upon Cu supplementation is likely a result of increases in lipase activity, growth hormone secretion, and expression of genes involved in post-absorptive metabolism of lipids. The growth-promoting effects of dietary Cu have also been attributed to its bacteriostatic and bactericidal properties because Cu may change bacterial populations in the intestine, and thereby reduce inflammation caused by pathogens. However, further research is needed to determine potential interactions between Cu and non-nutritive feed additives (e.g., enzymes, probiotics, phytobiotics), and the optimum quantity of Cu as well as the optimum duration of feeding supplemental Cu in diets for pigs should be further investigated. These gaps needs to be addressed to maximize inclusion of Cu in diets to improve growth performance while minimizing diseases and mortality.

## Introduction

Minerals are inorganic elements needed by pigs for maintenance, growth, and reproduction [1]. Historically, mineral nutrition of domestic animals was considered of limited importance with the exception of common salt, which was recognized in Biblical times as a substance of value for human and animal consumption [2]. Discovery of the essentiality of minerals dates back to the late eighteenth century when it was recognized that deficiency of minerals caused certain diseases [1]. The nutritional significance of minerals was demonstrated in 1791 when Fordyce demonstrated that canaries require an adequate Ca supply for optimum health and egg production [3], but most of the early research with minerals was conducted to alleviate health problems [3].

Minerals have structural, physiological, catalytic, and regulatory functions in animals [4] and they are classified into 2 groups based on the amount that is required in the diet. Minerals needed by more than 100 mg/kg diet on a dry matter basis are called macro minerals, and this group includes Ca, Cl, K, Mg, Na, P, and S [1]. These minerals play a major role in acid-base balance, structural and regulatory functions in bones and teeth, and nerve transmission. Minerals that are required in quantities less than 100 mg/kg diet are called micro minerals, and primarily serve as components of enzymes, coenzymes, and hormones [5]. Micro minerals include Cr, Fe, I, Mn, Mo, Co, Se, Zn, and Cu [6].

Copper (Cu) is an essential component of several metalloenzymes including cytochrome C oxidase, lysyl oxidase, cytosolic Cu-Zn superoxide dismutase (SOD1), extracellular Cu-Zn superoxide dismutase 3 (SOD3), monoamine oxidase, and tyrosinase [7, 8]. Copper, therefore, plays a role in oxidation-reduction reactions, transport of oxygen and electrons, and protection against oxidative stress [8, 9]. Copper is involved in metabolic reactions including cellular respiration, tissue pigmentation, hemoglobin formation, and connective tissue development [10, 11]. Copper has been recognized as an essential mineral since 1928 when it was demonstrated that Cu is needed for red blood cell synthesis in rats. Rats suffering from anemia were fed animal or vegetable based diets supplemented with ash and were able to recuperate from the disease. It was subsequently discovered that ash contained Cu sulfide [12]. This discovery led to research that demonstrated the essentiality of Cu not only for preventing microcytic hypochromic anemia, but also for maintenance and growth [12, 13].

The objective of this contribution is to review current understanding of digestibility, absorption, and metabolism of Cu, Cu deficiency, Cu toxicity, Cu bioavailability, and relationships between Cu and other nutrients. Effect of pharmacological levels of Cu on growth performance, gut microbiome, and intestinal health of pigs will also be discussed.

## Digestibility, absorption, and metabolism of copper

Mineral digestibility reflects the dissolution and absorption of minerals from the gastrointestinal lumen. Digestibility and absorption of minerals is difficult to accurately determine due to endogenous mineral secretions into the gastrointestinal tract via pancreatic juice, bile, and mucosal cells [14]. Digestibility of Cu and Zn is also difficult to assess due to interference of homoeostatic regulation, which normally limits absorption of these minerals when animals are fed beyond the requirement [15]. The digestibility of Cu for growing pigs range from 30% to 55% [15, 16], and the relatively low digestibility of Cu is due to antagonisms between Cu and other microminerals [17]. Low pH in the stomach may reduce digestibility of Cu by causing dissociation of inorganic salts of dietary Cu [18]. As pH increases in the small intestine, Zn and Cu can be trapped in insoluble hydroxide precipitates, rendering these minerals unavailable for absorption [19]. The dietary source of Cu also affects its digestibility in pigs [15]. Chelation of dietary trace minerals with proteinates (i.e., peptides, amino acids) improve apparent total tract digestibility and retention of Cu in pigs by preventing formation of insoluble complexes along the gastrointestinal tract [16, 20].

Copper is mostly absorbed in the upper gastrointestinal tract, particularly in the duodenum, but some Cu is absorbed in the stomach [21]. In non-ruminants, Cu is primarily absorbed through a transcellular saturable process [22], but Cu can be absorbed through solvent drag, which involves movement of Cu through the tight junction pores [23]. Solvent drag allows free mineral ions to be solubilized in water through water dipole-ion interaction. Minerals suspended in water can be absorbed when water passes through the pores within the protein meshwork forming the tight junction [23].

Copper exist in two forms of valency depending on its state of oxidation. Most dietary Cu is in the Cu^2+^ form, but for Cu to be absorbed, it must be reduced to Cu^+^, which is catalyzed by a Cu-reductase enzyme that is expressed by glands at the brush border [24]. This metalloreductase belongs to the Steap protein family, and is a ferrireductase that stimulates cellular uptake of Fe and Cu [25]. Following the reduction of dietary Cu^2+^ into Cu^+^, Cu^+^ crosses the apical membrane and enters the enterocyte through Cu transport protein 1 (CTR1). Copper transport protein 1, which has a high affinity for Cu, is the main Cu transporter in enterocytes. Copper transport protein 1 is present in most tissues with significant quantities in the liver [26] because of the high need for Cu in hepatic cells. The amount of CTR1 in the apical membrane decreases via degradation in endosomal compartments if Cu is in excess of the requirement [27]. Other Cu transporters involved in Cu uptake are Cu transport protein 2 (CTR2) and divalent metal transporter (DMT1), but their affinity for Cu is less than that of CTR1 [28]. The DMT1 is located mainly on the brush border and transports Cu, Fe, Zn, and Mn across the apical membrane [29]. Thus, CTR1, CTR2, and DMT1 are the transport proteins specifically involved in increasing cellular Cu concentration if the body is in need of Cu.

Upon uptake of Cu^+^ from the apical membrane, Cu^+^ is transferred to chaperone proteins [30]. Chaperone proteins are involved in maintaining homeostatic Cu concentration in the body, and are associated with specific metalloenzymes and other Cu-containing proteins [27]. One of the chaperone proteins delivers Cu^+^ to Cu/Zn-superoxide dismutase, which is an antioxidant enzyme. Another chaperone protein is the cytochrome C oxidase Cu chaperone (COX17), which transports Cu^+^ in the mitochondria to cytochrome C oxidase, which is involved in energy transfer from NADH or FADH_2_ to ATP [29]. Other chaperone proteins include antioxidant protein 1 (ATOX1), which delivers Cu through the cytosol to the Golgi apparatus of intestinal cells [31]. Copper is then transferred to the Cu transporting ATPase 1 protein (ATP7A), which can bind and translocate 6 Cu^+^ ions into the basolateral membrane [32]. This ATPase also sequesters excess Cu to avoid Cu toxicity [33]. At the basolateral membrane, Cu^+^ is then converted to Cu^2+^ via a Cu oxidase for release into the interstitial space.

The homeostatic regulation of Cu absorption primarily involves the action of specific transporters and chaperone proteins [34]. The rate of Cu absorption is influenced by the Cu status of the animal, and Cu digestibility may be increased if animals are Cu-deficient [35]. If animals are deficient in Cu, there is an increase in the synthesis of Cu transport proteins and a Cu-ATPase pump is used to move Cu across the basolateral membrane into the extracellular fluid [35]. If the Cu concentration of the animal is adequate, the amount of Cu transport proteins for uptake is low, and the liver can synthesize metalloenzymes and store Cu for future use. If dietary Cu is provided in excess of the requirement, enterocytes produce a sulfhydryl-rich protein called metallothionein, which binds to the freely ionized Cu. This results in a subsequent reduction of Cu absorption, which helps prevent Cu toxicity [36, 37]. Metallothionein binds other metals such as Zn and Cd [37, 38]. Supplementing animals with greater quantities of Cu increases gene and protein expression of Cu specific transporters and chaperone proteins [39] because high concentration of Cu triggers ATP7A to become more active in releasing Cu^+^ at a higher rate [40]. However, research is needed to determine how pharmacological concentrations of Cu modulate expression of Cu transporters and chaperone proteins at the transcription level as well as at the level of translation.

In the hepatic portal vein, most of the absorbed Cu^2+^ is bound to albumin and transcuprein [41] for transport to the liver, where it is taken up by hepatocytes as Cu^+^ using Cu reductase. The CTR1 protein then moves Cu^+^ across the hepatocyte cell membrane. For Cu to be transported from the liver to peripheral tissues, Atox1 delivers Cu to the transmembrane Golgi complex. Copper is then transferred to the Cu transporting ATPase 2 protein (ATP7B) [32]. The Cu bound to ATP7B can then be utilized to produce Cu-containing proteins for export from the liver. Most Cu in serum is contained in ceruloplasmin, which is the major protein carrier for export of Cu from liver to target organs [42].

Ceruloplasmin is involved in Fe metabolism by having ferroxidase activity, which catalyzes the conversion of Fe^2+^ to Fe^3+^ [43]. The biological role of ceruloplasmin in pigs was reported by Ragan et al. [44] who demonstrated the impact of ceruloplasmin on plasma Fe in pigs fed diets deficient in Cu. Deficiency of Cu resulted in reduced concentration of serum ceruloplasmin with a subsequent manifestation of anemia in pigs. Iron deficiency was only corrected by administration of homologous ceruloplasmin or Cu to Cu-deficient pigs [44]. Porcine ceruloplasmin can be classified as ceruloplasmin I or ceruloplasmin II [45]. Ceruloplasmin I has greater copper content and specific enzymatic activity compared with ceruloplasmin II. Newly born piglets typically have high concentrations of liver Cu with ceruloplasmin II as the predominant form of ceruloplasmin. As pigs grow older, the concentration of ceruloplasmin I increases whereas ceruloplasmin II concentration remains constant [45].

## Deficiency and toxicity of copper

Animals deprived of Cu develop critical dysfunctions and hypocuprosis [46–49]. Microcytic anemia is a sign of Cu deficiency due to its role in Fe metabolism, specifically in hemoglobin formation and development [47, 50, 51]. Ceruloplasmin, which functions physiologically as a copper-dependent ferroxidase to promote transferrin formation, is essential for the catalysis of Fe^2+^ to Fe^3+^ [42]. Pigs suffer from bone abnormalities and unusual leg conditions with various degrees of crookedness if dietary Cu is deficient because of deficiency in monoamine oxidase, which is needed for cartilage formation [48, 49]. Depigmentation, failure of hair keratinization, and cardiovascular disorders have also been demonstrated as signs of Cu deficiency [52, 53]. More than 60% of pigs fed Cu-deficient diets died from coronary artery disease [54] characterized by intimal lesions in muscular arteries of Cu-deficient pigs [55]. Integrity of arteries in the cardiovascular system relies heavily on the quality and quantity of collagen and elastin, and Cu-dependent oxidases (i.e., benzylamine oxidase and lysyl oxidase) are needed for collagen and elastin metabolism [56]. Pigs with hypocuprosis have impaired humoral response [57]. Copper plays an important role in the development and function of T and B cells, neutrophils, and macrophages [58, 59], and deficiency of Cu affects the immune system because of deficiency in cytochrome C oxidase and superoxide dismutase [57]. Low concentration of cytochrome C oxidase results in impairment of the respiration burst in neutrophils, and subsequently a decrease in immunological function [60].

The clinical signs and symptoms that are typically observed in pigs with Cu deficiency have always been associated with the role of Cu as a component of metalloenzymes needed for several metabolic reactions such as cellular respiration, hemoglobin formation, cartilage formation, and keratinization [48]. A reduction in growth performance and feed intake occurs when Cu is deficient in diets for all species; however, an unusual leg condition develops specifically in Cu-deficient pigs [61]. Pigs fed diets that are deficient in Cu have signs of central nervous system disorders such as ataxia, posterior paresis, and horizontal nystagmus, and these observations is possibly due to a deficiency of cytochrome C oxidase needed for phospholipid synthesis [58, 62]. Deficiency in Cu may be related to the degree of saturation of the animals’ lipid reserves and cholesterol profile because dietary Cu is believed to influence lipid metabolism in animals [63, 64]. Addition of increasing levels of dietary Cu as CuSO_4_ reduced the concentration of serum polyunsaturated fatty acids in pigs fed diets containing 5% fat as stabilized medium-chain triglycerides, whereas the concentration of serum polyunsaturated fatty acids increased in pigs fed diets without added fat [65]. Copper supplementation may also affect carcass fatty acid composition of pigs because supplementation of Cu in diets resulted in increased proportion of unsaturated fatty acids in the outer backfat, inner backfat, and perinephric backfat of pigs [66, 67]. Deficiency of Cu causes hypercholesteremia and hypertriglyceridemia [68], and the reason for these conditions has been attributed to the role of Cu in increasing lipoprotein lipase and triolein hydrolase activities [64]. The effect of Cu deficiency on accumulation of long chain fatty acids has been attributed to increased expression of fatty acid synthase with reduced concentration of ceruloplasmin in the serum [69] because Cu is needed for ceruloplasmin to function. As Cu concentration increases, and is greater relative to the requirement, ceruloplasmin activity increases [70], which inhibits lipid peroxidation by inhibiting glutathione peroxidase and catalase [71].

Pigs are less sensitive to Cu toxicity than ruminants [5]. Sheep and lambs can only tolerate a Cu level of 20 mg/kg and 100 mg/kg (dry matter basis), respectively [72, 73]. Differences in the tolerance level for Cu among species can be attributed to the capacity of the animal to excrete Cu in the bile, and in general, pigs excrete more Cu compared with ruminants [5]. Pigs also absorb Cu more efficiently compared with ruminants [74, 75]. If the dietary level of Cu is in excess of the requirement, Cu accumulates in the liver and other vital organs. This may result in increased concentration of unbound free ionized Cu, which is a strong oxidant leading to haemolysis [76]. In pigs, Cu can be toxic if more than 250 mg/kg of diet is fed for an extended period because this leads to hemolysis of red blood cells characterized by jaundice and necrosis [76, 77]. Inclusion of 750 mg of Cu per kg of diet in growing pigs resulted in increased Cu and aspartate transaminase (AST) concentrations in the serum [78], and the observed increase in serum AST concentration indicates damage to tissues where AST is abundant (i.e., kidney, liver) [79]. Signs of jaundice were observed in pigs when fed diets with 750 mg/kg of Cu, which was proposed to be a result of liver damage due to the relationship between the increased serum AST concentration and the degree of jaundice [78]. However, addition of 500 mg/kg of Zn or Fe to diets containing 750 mg/kg of Cu prevented clinical signs of copper toxicity and resulted in normal serum concentration of AST [78].

High concentrations of Cu in the diet could also promote lipid peroxidation in cell membranes by inducing oxidative stress in diets as well as in the body [80]. Lipid peroxidation causes degradation of unsaturated fatty acids, which results in a reduction of energy in diets, and as a consequence, could negatively affect growth performance and health of pigs [81]. One method to determine the degree of peroxidation in the animal’s body is through the use of malondialdehyde [82]. Malondialdehyde is commonly used as a biomarker of oxidative stress, and the thiobarbitoric acid assay is a method frequently used to determine malondialdehyde in biological fluids and tissues [83]. The degree of oxidative stress varies and is influenced by diet type and source of Cu. Dietary factors that act as antagonists for Cu absorption, such as high concentrations of Zn and phytate, alleviate the pro-oxidant effects of excess Cu [39]. The major sources of Cu fed to pigs include CuSO_4_ and Cu hydroxychloride, and these sources vary greatly in their chemical characteristics [84]. Pigs fed diets with 225 mg/kg Cu hydroxychloride had reduced duodenal malondialdehyde concentrations compared with pigs fed CuSO_4_ at the same concentration, which resulted in less oxidative stress in the intestine [39].

## Assessment methods for copper bioavailability

One way to evaluate efficacy of sources of Cu is to measure relative bioavailability or digestibility. Relative bioavailability of dietary Cu is defined as the proportion of the ingested dietary Cu that has been chemically absorbed and can be utilized by the animal for maintenance and growth [85]. Bioavailability is also defined as the proportion of an ingested nutrient that is absorbed, transported to its site of action, and utilized to synthesize a physiologically active metabolite [86]. Estimates for relative bioavailability of different Cu sources is commonly obtained through slope-ratio assays [87]. In this assay, diets with graded levels of Cu are formulated, and responses indicative of Cu status of the animals are evaluated [88]. Responses include tissue concentrations of Cu, concentrations of metalloproteins, or enzymatic activity of animals fed the test diets. The slope of the regression line obtained from animals fed the test source of Cu is compared with that from animals fed a reference Cu source [89, 90].

Cupric sulfate pentahydrate is the most commonly used reference standard for estimating bioavailability of Cu from different sources [91]. The relative bioavailability of Cu is evaluated *in vivo* using Cu radioisotopes and plethoric dietary supplementation [85]. Liver, bile, and gall bladder are usually harvested and Cu concentrations are measured to assess relative Cu bioavailability [92–94]. Plasma Cu concentrations, metalloproteins, and metalloenzymatic activities (ceruloplasmin, cytochrome C oxidase, and Cu-superoxide dismutase) can also be used as indicators of Cu status [95]. In pigs, Cu-Lys and Cu-Met are more bioavailable than CuSO_4_, whereas cupric carbonate and Cu citrate are less bioavailable than CuSO_4_ (Table 1). In general, plant feed ingredients are variable in the bioavailability of Cu and have lower bioavailability of Cu than animal and sources of Cu [85] because the majority of Cu in plant feed ingredients is bound to phytate [88]. However, Cu from pork liver has low bioavailability compared with other sources due to high concentration of Zn in the liver, which may inhibit Cu availability [88]. Copper oxide also has low bioavailability when fed to ruminants, poultry, and pigs [93, 96] due to the inability of copper oxide to solubilize in acidic conditions with relatively high passage rate in the gastrointestinal tract [95].

**Table 1:** Relative bioavailability of Cu sources for pigs^a^

Source	Relative bioavailability^b^
CuSO_4_	100
Cu-Met	110
Cu-Lys	112
Cupric carbonate	85
Cupric oxide	0
Cupric sulfide	10
Cu citrate	84

^a^Values were adapted from Baker and Ammerman [85]

^b^The relative bioavailability is expressed relative to the bioavailability of Cu in CuSO_4_

Enzyme efficacy, digestibility, and *in vitro* bioavailability of Cu in feed ingredients have been studied [97]. Results of an *in vitro* digestibility assay indicated that CuSO_4_ and Cu hydroxychloride were completely dissolved during stomach digestion simulation, but the solubility of Cu from Cu hydroxychloride was more influenced by the pH of the digesta than Cu from CuSO_4_ if fed to poultry [98, 99]. Copper from CuSO_4_ was completely dissolved at pH 6.8, 4.8, 3.0, and 2.0. In contrast, Cu from Cu hydroxychloride was not soluble at pH 6.8, but solubility gradually increased as pH decreased [100]. The concentration of Cu in diets may also affect its solubility in the stomach. Solubility of Cu during simulated stomach digestion in pigs increased if 250 mg/kg of CuSO_4_ or Cu hydoxychloride was included in a control diet that contained 15 mg/kg of Cu [99]. Due to the low concentration of Cu in the control diet, other feed ingredients inhibit dissolution of Cu. Therefore, supplementation of CuSO_4_ or Cu hydoxychloride to the control diet may have increased the proportion of Cu available for stomach dissolution in pigs [99].

## Copper sources and requirements for pigs

The Cu that is included in pig diets usually originates from plant or animal-based feed ingredients or from mineral supplements. Most commonly used cereal grains and their co-products in swine diets contain 4.4 to 38.4 mg/kg of Cu on an as-fed basis (Table 2), but the amount of Cu in each plant feed ingredient vary depending on the variety, type of soil on which plants grow, maturity stage, and climatic conditions during growth [18]. Oilseed meals including soybean meal, cottonseed meal, and linseed meal usually have greater Cu concentration compared with cereal grains [101]. Fermentation of plant feed ingredients increases concentration of crude protein and ash because soluble carbohydrates are fermented, and therefore, the concentration of Cu may also increase [102]. Animal protein sources commonly used in pig diets include fish meal, poultry meal, and blood meal and these ingredients are generally comparable in Cu concentration to plant feed ingredients ranging from 8 to 36 mg/kg [76]. Copper in milk products such as skim milk powder, lactose, casein, and whey powder ranges from 0.10 to 6 mg/kg [76].

**Table 2:** Copper concentration in feed ingredients^a^

Feed ingredient	Average Cu content (as-fed basis), mg/kg
Corn, white	4.4
Corn, yellow	4.7
Rye	5.9
Oats	6.8
Barley	7.2
Oat groats	7.6
Wheat	7.8
Fish meal	8.0
Rice bran	9.0
Wheat germ	9.0
Millet, Japanese	9.1
Wheat middlings	12.1
Meat and bone meal	11.0
Blood meal	13.1
Flaxseed meal	16.2
Brewer’s dried grain	16.4
Wheat bran	16.4
Wheat gluten	17.2
Cottonseed meal	21.8
Linseed oil meal	21.8
Soybean	22.7
Meat meal	23.1
Corn gluten meal	35.1
Poultry meal	35.7
Distillers dried gains with solubles	38.4

^a^All values were adapted from O’Dell [101] and NRC [76]

Supplemental Cu is provided by fortifying complete diets and premixes with Cu from CuSO_4_, copper chloride, Cu amino acid complexes, or Cu hydroxychloride. Copper sulfate pentahydrate (CuSO_4_·5H_2_O) is a blue crystalline Cu salt commonly used as a pesticide, fungicide, soil additive, and feed supplement [103]. Copper sulfate is soluble in water with decreased solubility upon subjection to increased acid conditions [104]. Copper sulfate is the most common form of supplemental Cu in animal feeding due to its availability, and its relatively low cost compared with other sources of Cu [105]. Results of a number of experiments have documented the effects of CuSO_4_ in enhancing growth performance and gut health in weanling pigs [106–109]. However, using pharmacological concentrations of CuSO_4_ in pig diets have resulted in antagonisms with other dietary constituents [110] and environmental concerns due to high excretion of Cu in feces [111]. Due to potential negative effects on the external environment, the European Union, China, and other countries recently reduced the authorized maximum concentration of Cu in animal feed [112]. Excessive use of Cu in diets fed to pigs resulted in acquired copper resistance in gram-negative bacteria (e.g., *Escherichia coli, Pseudomonas syringae*) and gram-positive bacteria (e.g., *Bacillus subtilis, Lactococcus lactis*), which may lead to antibiotic resistance and negatively influence antibiotic treatments for diseases [113–116]. Therefore, other forms of supplemented Cu, which are generally included in diets at a lower inclusion rate and are less reactive with other nutrients, have been introduced to the feed industry. Examples of other sources of Cu include chelated Cu and Cu hydroxychloride. Chelation involves binding of Cu to a ligand (i.e., ethylenediaminetetraacetic acid, hydrolyzed soy protein, amino acids, or polysaccharides), and it is possible that Cu from these sources is absorbed more efficiently and have higher retentions compared with Cu from CuSO_4_ [16, 112, 117]_._ Indeed, inclusion of chelated Cu in diets for weanling pigs is as effective as use of CuSO_4_ in improving growth performance [117–119]. Addition of 100 to 200 mg of Cu per kg complexed with amino acids such as CuLys is also as effective, and in some cases more effective, than Cu from CuSO_4_ in increasing average daily gain (ADG) and average daily feed intake (ADFI) in weanling pigs [120, 121]. In an experiment conducted by Ma et al. [122], treatments included 2 supplemental levels of Cu (50 or 250 mg/kg) and Cu from either Cu(2-hydroxy-4-methylthio butanoic acid)_2_ or CuSO_4_. Results indicated that Cu(2-hydroxy-4-methylthio butanoic acid)_2_ was more efficient than CuSO_4_ in improving feed efficiency [122]. Another source of inorganic Cu is Cu hydroxychloride, and several experiments demonstrate that this source of Cu, when included at 150 mg/kg, enhances growth rate and feed efficiency in pigs (Table 3). Copper hydroxychloride is insoluble in water due to covalent binding of Cu to hydroxyl groups, but it is highly soluble in acidic conditions, which makes it less reactive in vitamin-mineral premixes, less toxic, and it has less pro-oxidant activity than CuSO_4_ [84, 129, 130].

**Table 3:** Growth performance of pigs fed diets containing 0 or 150 mg Cu per kg from Cu hydroxychloride. Average of 7 experiments^a^

Item	Diet		SEM	*P*-value
	Control	Control + Cu^b^		
d 0 to 14				
Initial body weight, kg	9.641	9.629	1.552	0.745
ADG^c^, kg	0.319	0.376	0.082	0.002
ADFI^c^, kg	0.590	0.611	0.149	0.058
G:F^c^	0.557	0.636	0.046	0.015
Final body weight, kg	15.415	16.223	2.947	0.004
d 14 to 28				
ADG, kg	0.596	0.625	0.072	0.044
ADFI, kg	1.029	1.018	0.144	0.846
G:F	0.595	0.621	0.024	0.286
Final body weight, kg	22.675	23.857	4.111	0.001
d 0 to 28				
ADG, kg	0.458	0.498	0.021	< 0.001
ADFI, kg	0.807	0.814	0.154	0.817
G:F	0.589	0.623	0.026	0.046

^a^Experiments included from [123–128]

^b^The diet containing added Cu was fortified with 150 mg/kg of Cu from Cu hydroxychloride (IntelliBond C^II^; Micronutrients USA; Indianapolis, IN)

^c^*ADG* Average daily gain, *ADFI* Average daily feed intake, *G:F* Gain to feed ratio

The requirement for Cu by pigs is influenced by dietary factors and age of the animal. Neonatal pigs usually require 5 to 10 mg of Cu per kg of diet for normal metabolism [76, 131, 132] and as pigs get older, the requirement for Cu decreases. A requirement of 5 to 6 mg of Cu per kg of diet has been suggested for growing pigs [73, 76]. Both primiparous and multiparous sows require supplementation of 10 mg of Cu per kg of diet during gestation [49]. Limited information is available about feeding high levels of Cu for gestating and lactating sows, but including 60 mg of Cu per kg of diet for sows improve reproductive performance compared with sows fed a diet containing 6 mg/kg of Cu [76]. Sows fed diets containing 250 mg/kg of Cu from CuSO_4_ had reduced culling rate, farrowed larger litters of pigs, and had heavier pigs at birth and at weaning compared with sows fed diets without added Cu [133].

Dietary factors that interfere with Cu absorption, and therefore may influence the need for Cu, include dietary Zn, Fe, S, Mo, and phytate. Zinc is closely related to Cu, chemically and physiologically [134]. Zinc is an essential component and activator of several metalloenzymes, and some of these metalloenzymes, such as superoxide dismutase, has both Cu and Zn as one of its components [135]. High concentrations of dietary Zn increase the requirement for Cu [131] by inducing high concentrations of intestinal metallothionein, which binds Cu, and decreases Cu absorption [36]. High Zn intake, therefore, induces clinical signs of Cu deficiency [136–138]. High dietary concentrations of Fe decrease Cu absorption, which lead to Cu deficiency [139]. It is believed that Fe and Cu have antagonistic effects due to competition for absorption sites in intestinal mucosa [139], and the interference of Fe in Cu absorption involve formation of ferrous sulfide complexes [140]. The sulfide part in the complex forms insoluble complexes with Cu [141]. The presence of phytate in the diet can also affect Cu absorption because phytic acid binds dietary cations including Cu, rendering them unavailable for digestion and absorption [142]. Phytase supplementation, therefore, increases Cu absorption by releasing Cu from phytic acid [143], but microbial phytase may decrease Cu availability by releasing significant amounts of Zn bound to phytate [92].

## Growth promoting levels of copper

Supplementing Cu to diets fed to weanling pigs at 100 to 250 mg/kg improve ADG and ADFI [108, 144, 145]. Reduction in diarrhea frequency and increased feed efficiency were also observed when high concentration of Cu was included in diets for weanling and growing pigs [123, 146]. Addition of 60 to 250 mg of Cu per kg in sow diets during late gestation and lactation reduce pre-weaning mortality [147] and increase pig weaning weights [148], presumably because of increased milk production. The greater ADFI reported for pigs fed diets supplemented with Cu is possibly due to the role of Cu in upregulating the mRNA expression of neuropeptide Y [149], a neuropeptide considered a feed intake inducer [150]. Copper also stimulates the secretion of growth hormone releasing hormone [151, 152] and is important for post-translational modification of regulatory peptides [153]. One of the hypothesized mechanism of Cu in improving growth performance is that Cu may stimulate activities of enzymes involved in nutrient digestion [154]. Addition of high concentrations of Cu increased lipase and phospholipase A activities in the small intestine [155], which may result in increased absorption of fatty acids and improved growth performance. However, supplementation of Cu at 150 mg/kg in diets for growing pigs did not improve apparent total tract digestibility of energy or true total tract digestibility of fat [124, 125]. Inclusion of 45 mg of Cu per kg of diet improved body weight gain of rabbits by upregulating the mRNA transcription of fatty acid transport protein and fatty acid-binding protein (FABP), and carnitine palmitoyl transferase 1 [156]. Supplementation of Cu to diets increased lipogenesis and fatty acid uptake in fish, indicating that dietary Cu influences post-absorptive metabolism of lipids [157]. Copper supplementation in diets for finishing pigs did not affect mRNA transcription of intestinal CTR1 and FABP [158]. However, supplementation of Cu at 150 mg/kg in diets for growing pigs increased the abundance of lipoprotein lipase and FABP1 in the subcutaneous adipose tissue and liver, respectively [126]. Therefore, the observed improvement in growth performance of pigs fed the Cu-supplemented diets may be a result of improved lipid metabolism with a subsequent improvement in energy utilization [126].

The growth-promoting effects of dietary Cu have also been attributed to its bacteriostatic and bactericidal properties [109] because Cu may alter the bacterial populations in the intestine, and thereby affect the growth and community structure of microorganisms in the cecum and colon [159]. Copper alters the 3-dimensional structure of bacterial proteins, which prevents bacteria from performing their normal functions [160]. Copper may disrupt enzyme structures and functions of bacteria by binding to S or carboxylate-containing groups and amino groups of proteins [161]. A high-Cu diet did not improve growth performance of germ-free pigs, but the high-Cu diet increased ADG and ADFI in conventionally reared pigs [162]. *Clostridium, Escherichia coli,* and *Salmonella* viable counts were reduced in the small intestine, and the numbers of coliforms were reduced as well in the cecum and colon of pigs upon Cu supplementation [106, 107, 163]. Copper supplementation in weanling pig diets reduced the counts of enterococci in the stomach and increased the lactobacilli population in the cecum of young pigs [159, 164]. Reduction in concentration of lactate, short chain fatty acids, biogenic amines (histamine, cadaverine, and putrescine), ammonia absorption, and urease activity in the gastrointestinal content of pigs were observed if 175 to 250 mg of CuSO_4_ per kg was supplemented to diets for weanling pigs [159, 163, 165, 166]. Supplementation of 150 mg/kg Cu as Cu hydroxychloride in diets for growing pigs also resulted in a reduction in microbial protein concentration, which is likely due to the ability of Cu to inhibit growth of microbes in the intestinal tract of pigs [167].

Weanling pigs are susceptible to infections, diseases, and villous atrophy in the gut, which result in physiological and pathological changes and altered intestinal tight junction barrier resulting in increased intestinal permeability [168, 169]. Tight junctions are made up of integral membrane proteins, mainly occludin and zonula occludens protein-1, and the integrity of the tight junctions is one of the important components of the intestinal mucosal barrier function [170]. Intestinal permeability increases upon diarrhea, which allows entry of toxins and pathogenic microorganism through the epithelial cells [171]. Inclusion of Cu at 100 to 200 mg/kg in diets fed to weanling pigs increases villus height and reduces crypt depth, thus improving intestinal health [172]. A reduction in concentrations of plasma diamine oxidase and *D*-lactate was observed when diets were supplemented with Cu-exchanged montmorillonites at 1500 mg/kg [163]. Diamine oxidase is located exclusively in intestinal villus and its preference in blood plasma serves as a marker for mucosal injury [173]. When pigs undergo stress and intestinal mucosal barrier is damaged, intestinal mucosal cells are being sloughed into the lumen, which leads to increased concentration of diamine oxidase [163]. Plasma *D*-lactate is a byproduct of intestinal bacteria, and excessive production of this metabolite pass through the damaged mucosa [173]. Therefore, the observed reduction in plasma diamine oxidase and *D*-lactate upon supplementation of dietary of Cu to diets indicates reduction in intestinal permeability and improvement of intestinal health. However, this is in contrast with data indicating that supplementation of Cu hydroxychloride did not affect the lactulose:mannitol ratio in pigs [127] indicating that high concentration of dietary Cu did not impact intestinal permeability of pigs.

Copper plays an important role in improving the innate and acquired immune function of animals [174], but improvements in the immune status of pigs fed high-Cu diets may be indirect because of the bacteriostatic property of Cu, which may reduce inflammation caused by pathogens [123, 174]. Exposure of pigs to pathogenic or nonpathogenic antigens results in an activated immune system and subsequent release of cytokines such as tumor necrosis factor α, interleukin-1, and interleukin-6 [168]. Pigs fed diets containing 3000 mg of Zn per kg and 250 mg of Cu per kg had reduced plasma cytokine circulation after a coliform lipopolysaccharide challenge, which likely indicates that both Cu and Zn can reduce infection and alleviate stress responses induced by bacterial endotoxin [175]. Likewise, pigs fed diets containing nanoCu had greater ADG and feed efficiency, and greater concentrations of γ-globulin, total globulin protein, and IgG compared with pigs fed the control diet [176]. Supplementation of dietary Cu to diets also resulted in reduced tumor necrosis factor α concentration and increased activity of superoxide dismutase in blood serum of weanling pigs [123, 176]. This indicates that the observed improvement in growth performance in pigs fed the Cu-supplemented diets was possibly due to improved antioxidant capacity and humoral immune response, which can prevent susceptibility of pigs to infections and diseases.

## Conclusions

Copper is an important micronutrient needed for maintenance, growth, and optimum health. Inclusion of 75 to 250 mg/kg of Cu in diets for pigs improve feed intake and feed efficiency. Results of several experiments demonstrated that the consistent improvement in growth performance upon Cu supplementation to diets is likely a result of the ability of dietary Cu to modulate intestinal microbial populations, increase lipase activity, stimulate secretion of neuropeptide Y and growth hormone, regulate antioxidant system, indirectly improve the immune response, and increase mRNA abundance of genes involved in post-absorptive metabolism of lipids in pigs. Dietary factors that interfere with Cu absorption was discussed, but further research needs to focus on determining potential interactions of Cu with non-nutritive feed additives (e.g., enzymes, probiotics). The optimum amount and duration of feeding supplemental Cu in diets fed to pigs also need to be further investigated. By addressing these gaps in the knowledge about Cu, the use of Cu in the feeding of pigs can be optimized.

## Data Availability

Not applicable.

## Abbreviations

ADFI: Average daily feed intake
ADG: Average daily gain
ATOX1: Antioxidant protein 1
ATP7A: Copper transporting ATPase 1 protein
ATP7B: Copper transporting ATPase 2 protein
AST: Aspartate transaminase
CTR1: Copper transport protein 1
CTR2: Copper transport protein 2
COX17: Cytochrome C oxidase Cu chaperone
DMT1: Divalent metal transporter
G:F: Gain to feed ratio
SOD1: Cytosolic Cu-Zn superoxide dismutase
SOD3: Extracellular Cu-Zn superoxide dismutase 3
